# A Probabilistic Model in Cross-Sectional Studies for Identifying Interactions between Two Persistent Vector-Borne Pathogens in Reservoir Populations

**DOI:** 10.1371/journal.pone.0066167

**Published:** 2013-06-20

**Authors:** Elise Vaumourin, Patrick Gasqui, Jean-Philippe Buffet, Jean-Louis Chapuis, Benoît Pisanu, Elisabeth Ferquel, Muriel Vayssier-Taussat, Gwenaël Vourc’h

**Affiliations:** 1 INRA, UR346 Epidémiologie Animale, Saint Genès Champanelle, France; 2 INRA, USC Bartonella et tiques, Maisons-Alfort, France; 3 Muséum National d’Histoire Naturelle, UMR 7204 CNRS-P6-CERSP, Paris, France; 4 Institut Pasteur, Equipe des Borrelia, Paris, France; University of Minnesota, United States of America

## Abstract

**Background:**

In natural populations, individuals are infected more often by several pathogens than by just one. In such a context, pathogens can interact. This interaction could modify the probability of infection by subsequent pathogens. Identifying when pathogen associations correspond to biological interactions is a challenge in cross-sectional studies where the sequence of infection cannot be demonstrated.

**Methodology/Principal Findings:**

Here we modelled the probability of an individual being infected by one and then another pathogen, using a probabilistic model and maximum likelihood statistics. Our model was developed to apply to cross-sectional data, vector-borne and persistent pathogens, and to take into account confounding factors. Our modelling approach was more powerful than the commonly used Chi-square test of independence. Our model was applied to detect potential interaction between *Borrelia afzelii* and *Bartonella* spp. that infected a bank vole population at 11% and 57% respectively. No interaction was identified.

**Conclusions/Significance:**

The modelling approach we proposed is powerful and can identify the direction of potential interaction. Such an approach can be adapted to other types of pathogens, such as non-persistents. The model can be used to identify when co-occurrence patterns correspond to pathogen interactions, which will contribute to understanding how organism communities are assembled and structured. In the long term, the model’s capacity to better identify pathogen interactions will improve understanding of infectious risk.

## Introduction

Many research disciplines in ecology seek to decipher processes behind species co-occurrence (*e.g.* community ecology [1]; macro-ecology [2], [3]; parasitology [4]). In medicine and epidemiology, a growing number of studies are reporting simultaneous infections by microorganisms which can be pathogenic in many different mammal hosts (wild and domestic animals and humans [5]–[8]). Pathogen interactions are of crucial medical concern because they can alter host susceptibility, infection length and clinical symptoms. From an epidemiological point of view, interactions may modify infectious risk. For example, in *Drosophila melanogaster*, infection with the bacterial symbiont *Wolbachia* increases resistance to some natural pathogens of Drosophila, the RNA virus [9]. How different taxa of microorganisms assemble can indicate their interaction within individual hosts. However, positive or negative co-occurrence of microorganisms that are due to interactions must be distinguished from those merely due to confounding factors such as environmental, behavioural and physiological host susceptibility (*e.g.* age, sex, location, season [10], [11]). For example, apparent associations in humans between the agent of Malaria and helminth infections may be due to common social or environmental factors rather than a true biological interaction [12].

In host populations, interactions between two microorganisms are suspected when the probability of coinfection is not random once confounding factors have been taken into account. Longitudinal studies, where host individuals are recaptured several times, allow the sequence of infection to be investigated to test whether infection by a given microparasite taxon modifies the infection probability by a subsequent one. For example, Telfer *et al.* [13] used a Generalised Linear Mixed Models (GLMM) to quantify the statistical interactions between the occurrence of 12 pairs of microorganisms in field vole populations. Such an approach is limited to infectious agents which can be detected without euthanizing the hosts, such as skin or blood microorganisms [11]. In cross-sectional studies, where many microorganisms can be detected, two main approaches are used. First, exploratory statistics investigate whether associations between taxa are statistically significant. The most popular method is the Chi-square test [14] because no *a priori* knowledge on the biological data studied is needed, and it is easy to implement [15]. To discriminate real biologic interactions from statistic associations, an improved Chi-square test has been further developed to take into account confounding factors (see [10]). In this study, Hellard *et al.* have applied their improved Chi-square test to four feline viruses: the Feline Immunodeficiency Virus (FIV), the Feline Herpesvirus (FHV), the Feline Calicivirus (FCV) and the Feline Parvovirus (FPV). However, Chi-square tests are not very powerful and do not give any details on the way an interaction works, *i.e.* the direction of the interaction or its intensity. Other exploratory approaches have been developed to detect statistical associations such as multivariate analyses (PCA, FCA) [16] and the network methods [17], which allow an overview of complex systems and can account for the organization of species interactions with each other. The second main approach used in cross sectional studies is based on the explanatory modelling of processes. The most well-known example of this type of approach is deterministic modelling (*e.g.* SIR or Susceptible, Infected and Recovered models) which allows several processes (demography, transmission, immunity) to be studied and dynamic data to be explored. The main drawback of is that such models require numerous parameters, many of which are poorly known and difficult to obtain. The models consequently are often associated with simulation models [18].

We propose a new modelling approach to identify potential pairwise interactions in cross-sectional studies that combines a probabilistic and a statistical model and allows testing counfouding factors. The statistical model with observed data is used to estimate the probabilistic model parameters. Our approach is built on the biological characteristics of microorganisms regarding transmission, persistence and pathogenicity. We developed our model to study two vector-borne pathogens that are considered persistent and non-pathogenic for their reservoir host. We applied the model to detect potential interactions between *Borrelia afzelii* and *Bartonella* spp. [19] in bank voles (*Myodes glareolus*). These two bacteria are borne by ticks (*Borrelia afzelii*) and fleas (*Bartonella* spp.), are pathogenic for humans, and are carried asymptomatically by rodents [20], [21]. They first colonize the dermis of their host, which is the interface between their host and flea and tick vectors [22], [23]. The two bacteria then use similar strategies to circumvent host defences, for example, stimulating an increase in the host’s production of interleukin to weaken its immune system [24], [25]). It is therefore suspected that the bacteria directly interact at the level of the dermis, and indirectly interact with each other through the immune system.

## Materials and Methods

### Overall Strategy

We used a multi-step approach to model interactions between the two vector-borne pathogens. First, we wrote a probabilistic model based on explicit biological hypotheses regarding the probability of infection by a microorganism, with or without the presence of a second microorganism, in juvenile and adult populations of the reservoir host. Second, we built a statistical model to estimate and test different parameter sets. Third, we compared the sensitivity of our model with a Chi-square test of independence by simulation. Fourth, we applied our model to cross-sectional data obtained in a reservoir population of bank voles (*Myodes glareolus*) coinfected by *Borrelia afzelii* and *Bartonella* spp.

### Probabilistic Model

**Figure 1:**
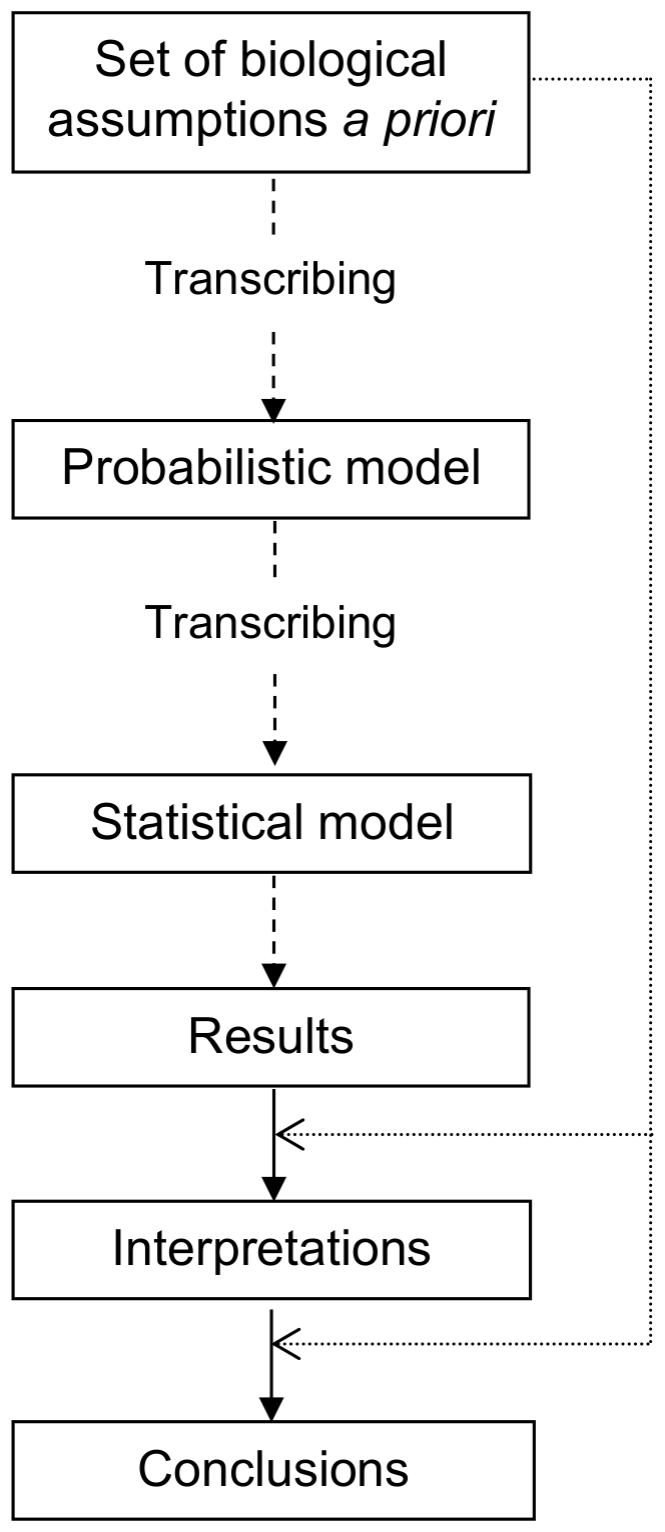
Schema of our approach to modelling interaction.

The probabilistic model set down the assumptions of a biological model (Figure 1). The assumptions were as follows: microorganisms (1) are detected correctly in hosts, *i.e.* the detection is sensitive (all infected individuals are detected) and specific (all non-infected individuals are found not infected); (2) are not vertically nor directly transmitted among host populations; (3) are persistent in reservoir hosts and (4) do not induce mortality. The life of an individual host was modelled with a juvenile and an adult age class. We assume that the studied population is in a dynamic equilibrium (birth-death).

**Figure 2:**
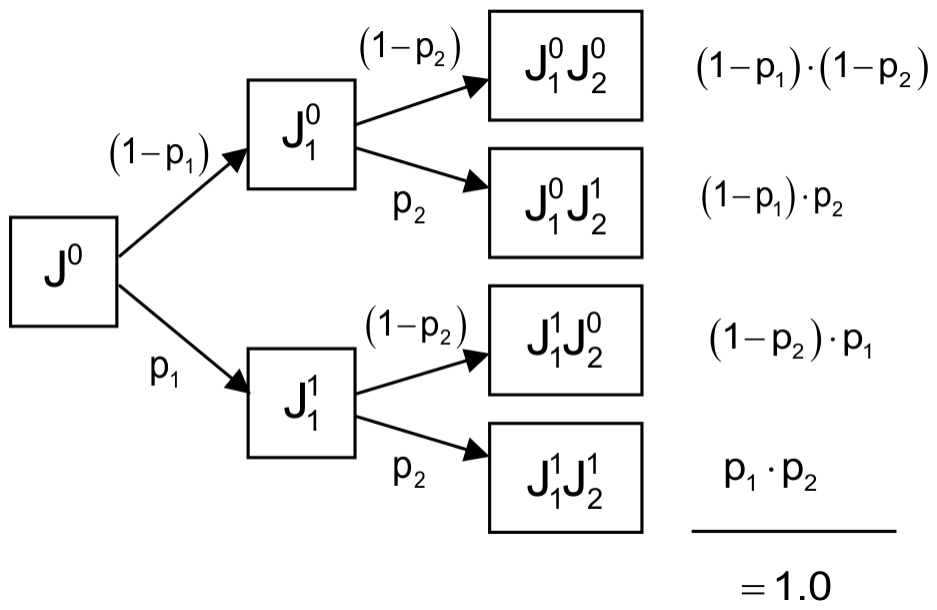
Model of the probability of microorganism infection of juvenile under independence hypothesis. 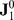
 indicates state of non-infection with microorganism 1 and 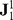 infected state with microorganism 1. It is the same for the microorganism 2. The probability of being infected with microorganism 1 (respectively 2) was defined with 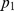 (respectively 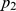). We assume the independence assumption of two microorganisms, the absence of vertical and direct transmission absence (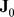 is initial state: free of any infection), the persistence of infection and the asymptomatic character of pathogens for reservoir host.

**Figure 3:**
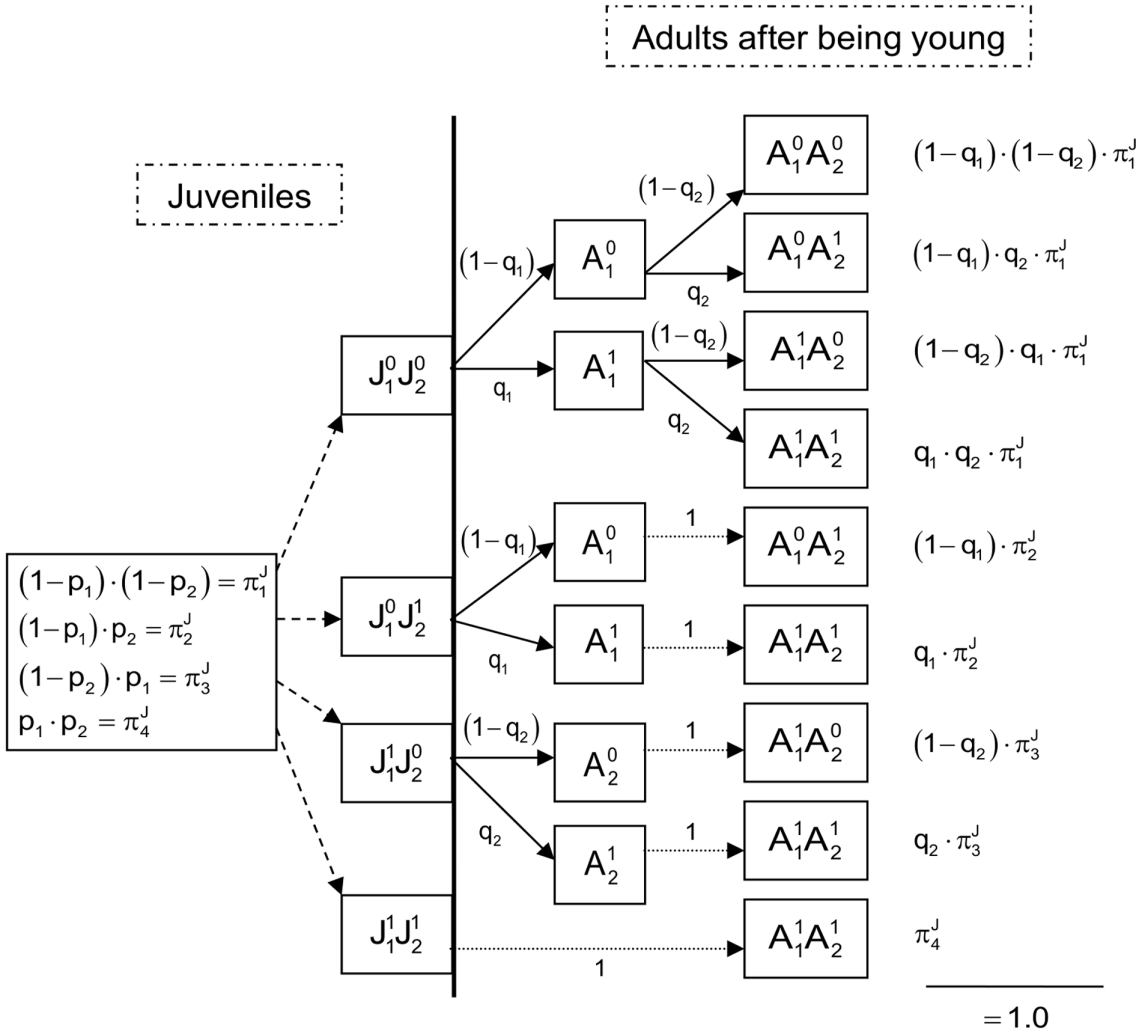
Model of the probability of microorganism infection of adult under independence hypothesis. 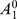
 indicates state of non-infection with microorganism 1 and 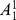 state infected with microorganism 1. It is the same for the microorganism 2. The probability of being infected with microorganism 1 (respectively 2) was defined with 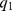 (respectively 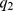). We assume the independence assumption of two microorganisms, from the final state of the Juvenile model, the absence of vertical and direct transmission, the persistence of infection and the asymptomatic character of pathogens for reservoir host.

Infection probabilities of two microorganisms in juvenile and adult populations were considered. The infectious states of individuals were noted as follows: 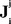, 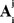, where **J** = Juvenile stage, **A** = Adult stage, **i** for the microorganism number (**i** = 1 for microorganism 1, **i** = 2 for microorganism 2) and **j** described the infectious state of the animal (**j** = 0 for uninfected, **j = **1 for infected). There were thus four probabilities **π** to be in an infectious state for each age stage, namely 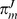 and 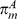 in juvenile and adult populations respectively, with **m** corresponding to the infectious state: 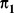 (0,0) not infected, 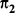 (1,0) infected by the first microorganism, 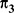 (0,1) infected by the second, and 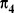 (1,1) infected by both (Tables 1 and 2). We modelled the probability to go from one infectious state to another. The probability of a juvenile to become infected by only one of the parasites was 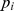 (Figure 2 and Table 3). Since we considered no vertical transmission, the initial infectious state of juveniles was always 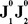. Second, the probability of an adult becoming infected by only one of the parasites was 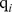, knowing that it was non-infected by the other microorganism during the juvenile state (Figure 3 and Table 4). Different infection probabilities between juveniles and adults were considered because the infection probability may vary due to such phenomena as maturation of the immune system (*e.g.* [26]). Each probability of being infected was detailed through conditional probabilities, *i.e.* the explicit equations of the model (Figures 2 and 3, Tables 3 and 4).

**Table 1:** The four 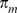 probabilities of infections between two microorganisms for juvenile state.

		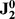	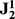
		*i = 2, j = 0*	*i = 2, j = 1*
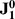	*i = 1, j = 0*	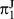	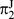
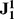	*i = 1, j = 1*	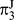	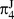

The parasite studied is referenced by i and j which describe the infectious state of the animal (0 = uninfected, 1 = infected), for more details see text.

**Table 2:** The four 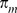 probabilities of infections between two microorganisms for adult state.

		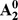	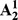
		*i = 2, j = 0*	*i = 2, j = 1*
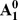	*i = 1, j = 0*	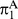	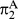
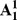	*i = 1, j = 1*	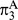	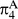

The parasite studied is referenced by i and j which describe the infectious state of the animal (0 = uninfected, 1 = infected), for more details see text.

**Table 3:** Probabilities of each microorganism infection event under non-independence in juveniles.

	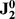	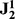
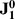	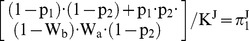	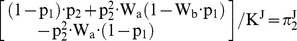
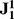	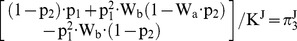	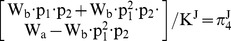

With 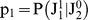, 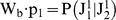, 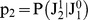, 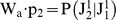 and 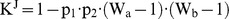, for more details see text and Table 1.

**Table 4:** Probabilities of each microorganism infection event under non-independence in adults.

	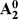	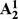
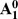	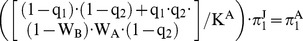	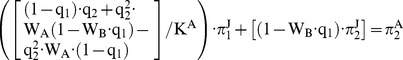
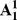	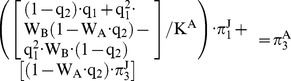	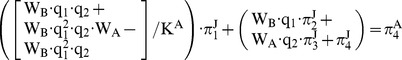

With 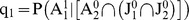, 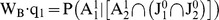, 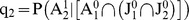, 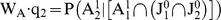 and 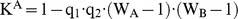, for more details see text and Table 2.

To model the interactions between microorganisms, an installation weight **W** was introduced that represents how the presence of a microorganism may modify the probability of infection by a subsequent microorganism. During the juvenile state, 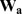 was the installation weight of parasite **1** on parasite **2** and 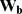 was installation weight of parasite **2** on parasite **1**.

If both infection probabilities were independent, 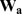 **_= _**1 and 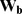 = 1. If 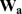 >1 and 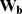 >1, each parasite had a facilitating role in the installation of the other. Conversely if 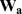 <1 and 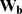 <1, each parasite had an inhibitory role in the installation of the other. Thus, the closest W was to 1, the weakest the interaction was. Through conditional probabilities, the complete probabilities system of each event for juveniles was obtained (see Table 3). The link that connects 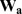 and 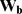 was highlighted by the equation (1) for the case of two microorganisms 1 and 2:



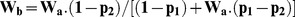
(1)In the same way, for adults 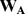 was the installation weight of parasite **1** on parasite **2**, and 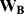 the installation weight of parasite **2** on parasite **1**. The system of conditional probabilities, as well as the link between 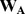 and 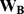, taking into account the infection state of individuals as juveniles, is shown in Table 4.

Altogether, there are eight infection states (four for juveniles and four for adults) in the model. The number of individuals studied, 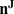 for juveniles, 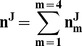, and 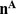 for adults, 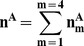, were considered as fixed because we worked with observed data. Translating this idea into a probability, we got 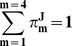 and 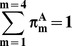. Since two parameters were known, six degrees of freedom were available. Therefore, all eight numbers of individuals 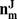 and 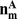 or eight probabilities of infections 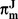 and 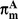 could be estimated with six parameters, 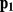,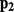,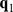,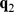, 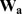 and 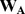 (see Tables 3 and 4).

In our model, the hypothesis of independence of both infections from each other is similar to that of the Chi-square test of independence hypothesis.

### Statistical Model

The probabilistic model was translated into a statistical model so that model parameters (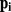,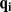,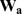 and 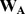) could be estimated, and the significance of different parameter sets (*i.e.* submodel or SM) and risk factors were tested (Figure 1). Different submodels were compared with the general model (GM) through likelihood ratio tests (Table 5). The general model was the model which gathered all parameters together. Risk factors that could favour the infection of both microorganisms were tested by writing submodels that considered risk factors as parameters. The final model was identified as the most parsimonious model *via* the AIC criteria [27] and the parameters of the final model were estimated.

**Table 5:** The different submodels compared to the general model.

Model Name	Parameter vector (number)
General Model	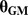 : p_1_, p_2_, q_1_, q_2_, W_a_, W_A_ (6 parameters)
Submodel 1 « W_a_ = 1 »	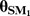 : p_1_, p_2_, q_1_, q_2_, W_A_ (5 parameters)
Submodel 2 « W_A_ = 1 »	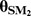 : p_1_, p_2_, q_1_, q_2_, W_a_ (5 parameters)
Submodel 3 « W_a_ = W_A_ »	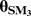 : p_1_, p_2_, q_1_, q_2_, W_a_ (5 parameters)
Submodel 4 « W_a_ = W_A_ = 1 »	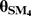 : p_1_, p_2_, q_1_, q_2_ (4 parameters)

The probability of being infected by a microorganism for juveniles is noted p and q for adult hosts. W_a_ is installation weight of parasite during an infection to juvenile state and W_A_ during an infection to adult state (for more details see text).

#### Likelihood of model

The general model was expressed as a multinomial distribution. The probabilities of each of the eight possible infection states depended on a vector of six parameters 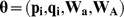 which were estimated by maximum likelihood. The parameters were constrained since probabilities of infection had to be between 0 and 1. Thus, 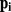 and 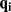 were defined in the interval 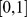 and 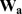 applied to a prevalence 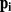 was defined in the interval 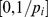 (it was the same for adults). In order to make the constraint implicit, the following transformations for parameters 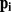 and 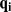 were performed: 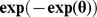 which was included in 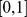 and 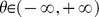. Similarly for 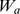 and 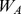, using 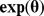 which was a positive value, when 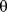 is in (–∞,+∞). In this way, the parameter vector 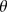 was estimated.

#### Testing submodels

The general model with the six parameters was a saturated model. In saturated models, estimated data are expected to match exactly to observed data. If they do not, then either some of the hypotheses are not relevant or an important factor influencing infection probability has been omitted. When this is the case, either a new model has to be constructed with additional parameters that translate the new hypotheses, or the missing factors have to be considered.

Since the different submodels were nested (see Table 5), likelihood ratio tests were used to test the significance of different submodels. The likelihoods of submodels were named **L_SM_** and were compared to the general model whose likelihood was **L_GM_** [28]. The test statistic was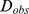.
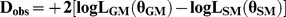



Under H0 (*i.e.* null hypothesis), the submodel should not be significantly different from the general model. Thus, the 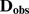 random variable followed a law of 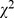, with 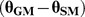 degrees of freedom, where 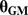 contained the parameter number of the general model and 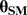 the parameter number of the submodel.

In the general model, none of the parameters were fixed, unlike those in the submodels. In fact, submodels 1, 2, 3 and 4 (Table 5) allowed us to test, by fixing the weights **W** of adults or juveniles to 1, whether the weights of infection were significantly different from one, *i.e.* whether associations of microorganisms were random. The possibility that the weight of infection varied according to the state (juvenile or adult) also could be tested. For example, in submodel 4 (see Table 5), microorganisms were randomly associated, and all interaction weights were equal to one. Microorganisms were randomly associated during the juvenile stage (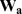
_ = _1) in submodel 1 and during the adult stage in submodel 2 (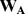
_ = _1). The weight of interaction was the same at the juvenile and adult stages in submodel 3. If a different biological model was used, other submodels could have been tested.

#### Estimating the final model confidence interval and parameters

Once the appropriated submodel, *i.e.* the final model, had been selected, the parameters of this model, *i.e.* probabilities and estimated weight of interactions 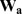 and 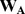 could be estimated. To do so, the inverse transformation on estimated parameters was performed. Thus 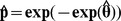 and 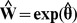.

With Hessian matrix inverse, the matrix of variance/covariance 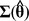 was estimated. Given that estimates of maximum likelihood 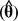 were Gaussian, the final confidence interval (CI) was calculated from the estimates and standard errors of the parameters 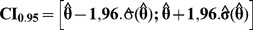 *or* 
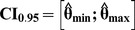
.

The parameter estimates and the 95% confidence intervals thus associated: 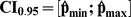 or 
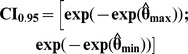
 and 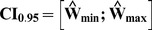 or 
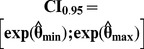
 were estimated in the original scale.

Then 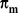 probabilities (*via* Tables 1, 2, 3 and 4) and expected values for the eight observed infection possibilities (four for juveniles and four for adults) were estimated.

#### Estimating risk factors common to microorganisms

Factors that may influence the risk and the weight of the infection were tested in the final model. The number of risk factors that can be tested depends on the degrees of freedom available. The effect **γ** of factors 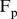, which can impact the probabilities of infection, was integrated as follows: 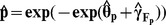, with 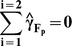. Similarly, the effect **γ** of factors 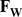, which can impact the weight of interactions, was integrated as follows: 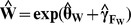, with 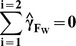.

All of the programs used in the analysis were written using R software (version 2.12.1) accessible on the site http://cran.r-project.org/. We used in particular the *nlm()* minimization function. We are in the process of developing a correctly assembled package of programming codes to facilitate its future use. All the functions that we used are available in a non-definitive form in the supplementary information (Functions S1).

### Simulation Study of Our Model *versus* Chi-square Test of Independence

Our model was compared to the Chi-square test of independence by simulating the sensitivity 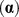 and power 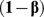 of both. The 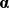 risk (or the error of second type) was the risk to conclude that the interaction (alternative hypothesis H1) was significant when in fact the association was random (null hypothesis H0). The 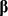 risk was the risk to conclude that the association was random (H0) when there was interaction (H1). The objectives were to verify that the 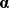 risk was well controlled *a priori* and to evaluate the influence of sample size on 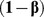 the power of tests. The statistic Chi-square test of independence for two microorganisms infecting juvenile and adult hosts, under the hypothesis H0, was considered. The estimated number 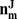 or 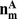 of each **m** modality of each state must be greater than five to perform this test. The distribution of the sensitivity 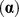 and the power 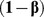 was simulated for both models according to the parameters and sample size. The dataset of 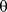 parameters and total sample size 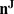 and 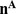 fixed *a priori*, were considered with the probabilities of juveniles and adults to become infected 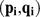 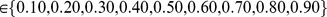, 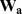 and 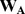 varied from 0.5 to 1.5 and the sample size varied from 200 to 1000.

### Biological model: application to infection of a bank vole population by *Borrelia afzelii* and *Bartonella* spp

#### Ethic statement

All conducted experiments complied with the current laws of France. Trapping and collection of rodents conducted on the study site (Forêt de Sénart, Essonne, France, 48°39′N, 2°29′E) were carried out under the control of Laurent TILLON (Office National des Forêts), Head of Research Group mammals. The project was approved by the Ethics Committee in Animal Experiment (CEMEA Auvergne). Steps were taken to ameliorate suffering in accordance with the recommendations [29]. Rodents were euthanized by cervical dislocation. Ear punch biospsies were limited to the minimum size needed, the puncture was disinfected with hydrogen peroxide and checked before releasing the animal.

#### Biological model

A total of 443 bank voles were tested, 252 being infected by *B. afzelii* and 49 being infected by *Bartonella* spp. PCRs were used to detect *B. afzelii* DNA on ear biopsies and *Bartonella* spp. DNA on liver and spleen samples [19], [30]. Both pathogens are considered asymptomatic in their rodent hosts [31]. They are not transmitted vertically or directly, so individual infections are considered to be independent [32], [33]. The age of the rodents was estimated according to their body mass. The individuals weighing less than 18 g were considered as juveniles (18 g being the smallest observed weight for a female in early gestation, J.-L. Chapuis, pers. comm.).

For *Borrelia afzelii*, we took samples from the ear, which, with its thin and highly vascularised epidermis, is highly appreciated by ticks and thus a prime site of *Borrelia afzelii* infection [34]. For *Bartonella* spp., we used a homogenate of liver and spleen. As the infecting *Bartonella* spp. are intraerythrocytic, not haemolytic, and these organs are “cemeteries of red blood cells”, this method confirmed our hypothesis of absence of false negatives [35]. The PCRs carried out are considered to be a specific detection method for both *B. afzelii* and *Bartonella* spp. [36], [37].

Both pathogens can also be considered persistent. Indeed, for *B. afzelii*, important mechanisms of immune escape in this bacteria (*e.g.* proteins provide protection from innate and adaptive host immune responses) seem to confirm the hypothesis of an on-going infection in rodents [24], [38], [39]. For *Bartonella* spp., infection is persistent in appearance. This intracellular bacterium is found mainly in red blood cells which have an average life of 30 days in rodents [40]. The bacterium is eliminated with the natural death of the cell. However, reinfection from infected vectors occurs constantly [41]. In addition, no resistance development (acquired immunity) has been shown *in vivo* [42]. The duration of the infection status is on average two months but infections overlap [43].

*Borrelia afzelii* was identified as microorganism 1 and *Bartonella* spp. as microorganism 2. Table 5 presents the first four submodels tested. Based on results and data, we then built and tested a fifth submodel in which « 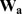 = 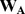 = **1** and 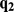 = 0 ». Only 3 parameters had to be estimated for submodel 5: 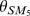: 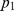,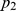,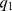. This submodel was tested because, in the data, the proportion of infected juvenile by *Bartonella* spp. seemed to be similar to that of infected adults. The influence of two risk factors on the model parameters 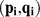
was tested: the presence of ticks on each rodent and the sex of the animal. These were chosen because both pathogens can be transmitted by ticks even though ticks are not the main vector for *Bartonella* spp. [44]. The sex of bank voles furthermore induces differences in their physiology and behaviour which have been shown to influence infectious risk probability [45].

## Results

### Simulation Results

For both modelling approaches (the probabilistic model and the Chi-square test of independence), the 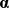 risk, under H0, even with a strong sample size gradient (**N**), varied little around the value of 0.05 (see respectively Table S1 and Table S2). In contrast, the power of the test 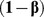, under H1, increased with the sample size. The more the infection weight (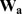 or 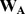) departed from 1, the lower the sample size could be for the power of the test to be maximal, *i.e.* 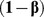 near 1 (Table S3 and Table S4). The reliability of detection of weak interactions depended on the sample size. In all cases, the probabilistic model was more powerful than the Chi-square test of independence, particularly to detect inhibition.

### Parameters of the Model for *Bartonella* spp. and *Borrelia afzelii* Coinfection in the Bank Voles

The general model, with the six parameters: 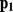,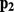,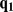,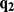,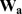 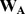, described perfectly observed data (*i.e.* the numbers of individuals in each infectious state are identical between estimated data and observed data). None of the submodels were significantly different from the general model (submodel 1, *P-value* = 0.064; submodel 2, *P-value* = 0.403; submodel 3, *P- value = *0.076; submodel 4, *P-value* = 0.179, submodel 5 *P-value = *0.328). However, with the AIC criteria of the submodels were the following: submodel 1, AIC = 904.305; submodel 2, AIC = 901.568; submodel 3, AIC = 904.026; submodel 4, AIC = 902.314; submodel 5 AIC = 900.314. Thus, submodel 5 (with 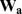 = 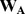 = 1 and 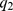 = 0) was the model that best fit the observed data (*i.e.* the smaller AIC criteria) and had the fewest parameters to estimate. As submodel 5 was the most parsimonious model, it was chosen as the final model.

In the final model, the values of different parameters describing the infection probabilities for *Borrelia afzelii* were 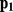: 0.017 *[0.003; 0.055]* and 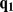: 0.130 *[0.090; 0.179]*, and for *Bartonella* spp.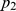: 0.569 *[0.521; 0.614]* and 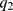: 0.000. Interaction weights between these two microorganisms were equal to 1 (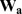 = 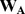 = 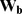 = 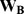 = **1**). Thus the probabilities of infection by both microorganisms were independent. These parameters allowed us to estimate the number of juveniles and adults for different infection states (see Table S5).

The probabilities of being in one of the infectious state were not significantly influenced by sex (*P-value* = 0.338) or tick carriage (*P-value* = 0.105).

The Chi-square test of independence, with 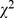 = 2.71 (*P-value* = 0.099) for juveniles and 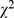 = 0.01 (*P-value* = 0.920) for adults, also indicated that both of the microorganisms’ distributions were independent.

## Discussion

Identifying when pathogen associations correspond to biological interactions is one of the challenges of multi-pathogen studies in populations. We propose a probabilistic modelling approach to identify potential pathogen interaction in cross-sectional studies. Our approach is based on explicit biological hypotheses (microorganism transmission and persistence characteristics) and is more powerful than the commonly used Chi-square test. The model we propose has the advantage of describing the observed data perfectly (*i.e.* the numbers of individuals in each infectious state are identical between estimated data and observed data) by using a probabilistic model with defined limits. We model the life of the host individual by differentiating the juvenile state from the adult state. Our model quantifies and characterises the direction of potential interactions, providing that relevant confounding factors are taken into account. This type of model can be used to describe the distributions of different species of pathogens in a reservoir host.

### Development of Our Approach and Comparison with Chi-square Test

Our modelling approach has been used little in epidemiology to date (Figure 1) [46]. The approach is based on the transcription of explicit biological hypotheses into a probabilistic model which is, in turn, transcripted into a statistical model. This approach has been used in plant [47] and medical research; for example, Bergemann *et al.* [48] applied it to assess neurocognition in various contexts. The main advantage of basing a study on *a priori* knowledge drawn from a biological model is that more precise conclusions can be obtained, and therefore mechanisms (*e.g.* interaction) can be characterized better. In contrast, basic statistical models such as Chi-square have fewer assumptions and thus the amount of information given by the models is reduced. With our method, if observed data did not correspond to the estimated data, it meant that a biological hypothesis posed *a priori* was not valid. Our approach thus involves a back and forth process between hypotheses and models. When the observed data correspond to the estimated data, the hypotheses cannot be rejected.

The model has several other advantages compared to a conventional Chi-square test. First, it is more powerful and the alpha risk (or the error of second type) is well controlled whatever the sample size (see Tables S2, S3, S4, S5). Second, the sample size in each category of individuals can be smaller than five, which is not the case for the Chi-square test. Third, our modelling strategy is based on transmission modelling between two life stages, juvenile and adult. That allows us to estimate the effect the infection of the first microorganism has on the infection of the second, and to quantify and describe the direction of potential interactions. Furthermore, this mimics what can be done with longitudinal studies using population data such as that conducted by Telfer *et al.* [13], in which generalized linear mixed models (GLMM) were employed. Finally, risk factors can be included easily in the different parameters of the models to discriminate true interactions from purely statistical interactions [10], [13]. However, the number of risk factors is limited since this number depends on the available number of degrees of freedom. In the example we provided, we could only study four categories of risk (male/female, infested by ticks/not infested) to avoid the over-parameterization of the model.

Our model has several limitations nonetheless. Our modelling approach requires an important amount of data for the detection of weak interactions, which is sometimes difficult to obtain (see Table S3). Indeed, the power of detection of interaction increases with the sample size, as is the case for other methods such as the Chi-square test. Furthermore, the sensitivity study of our model showed a better detection of facilitation than inhibition. This is because the infection weight (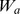 or 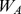), is bounded between 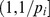 during facilitation, while its range is smaller (0,1) during inhibition.

### Application to *B. afzelii* and *Bartonella* spp. Data in a Bank Vole Population

Applying the model to *B. afzelii* and *Bartonella* spp. infection probabilities in bank voles, the presence of the first or the second bacterium did not influence the probability of infection of the other bacterium. Thus, no interaction was found, either directly or indirectly *via* the immune system. At their point of entry, the bacteria may have a limited opportunity to interact directly because they are inoculated at different locations on the host. Ticks, which transmit *B. afzelii*, are found most frequently on parts of the cephalic region where the skin is thin and unattainable by grooming (*i.e.* eyes, muzzle, ears), whereas fleas, which transmit *Bartonella* spp., have no preferred biting sites [49], [50]. However, despite different entry points, interaction through a serological cross-reactivity has already been found for *Borrelia burgdorferi* interacting with *Treponema pallidum* [51].

The probability for adults to be infected by *B. afzelii* (13%) was higher that the probability for juveniles (2%), a result commonly found in rodents [39], [52]. Surprisingly, the probability for rodents to be infected by *Bartonella* spp. at the adult stage was null. According to the sets of biological hypotheses in our model, this result means that most individuals were infected at the juvenile stage and not at the adult stage. Part of the population thus could be resistant to flea infestation, as was observed by Hawlena *et al.* [53] in other rodent and flea species. Another possible reason is that part of the population is resistant to *Bartonella* spp. infection, similar to what Greene *et al.* [54] observed in cats, where the cats became resistant to reinfection by *B. henselae*. However, the biologically surprising results obtained for adults force us to reconsider the hypothesis of persistence, which was based on the successive colonisation of hosts by different *Bartonella* species [55]. Another model without an assumption of persistence therefore should be developed.

None of the risk factors (tick carriage and sex) tested were significant. The absence of effect of sex on infection probability has been found in other studies. For instance, Bajer *et al.* [45] found that sex played a minor role for several haemoparasites, including *Bartonella grahamii*. The fact that we did not find any effect of tick carriage is rather surprising for *B. afzelii* [56]. The reason might be that the test lacked power because only presence/absence of ticks could be tested, not the quantified tick burden. Testing the impact of flea burden on infection probability of *Bartonella* spp. would have been interesting [33]. Unfortunately, the fleas were not harvested in our bank vole population.

### Perspectives: Developing other Probabilistic Models for other Sets of Biological Hypotheses

Our probabilistic model was specific to a certain sets of biological hypotheses, in particular to microparasites that were persistent, vector-borne and not pathogenic to the reservoir host. Two main hypotheses could be investigated to broaden the study of pathogen interactions to non-reservoir hosts and non-persistent microorganisms. The general approach would remain the same, but new parameters would be added, which would decrease the degrees of freedom. For instance, in the case of non-persistence, the model would have to take time into account. The probabilities of transition from one state to another would have to be estimated based on observed data.

Our paper proposes a novel approach to model the dependence of co-occurrence between two pathogens and to characterise this dependence. This methodology provides an opportunity to study interactions using data of cross-sectional studies, and this, despite the limited information available in the dataset (*e.g.* no information on infection span, no individual history). Our methodology is a population-centric modelling approach (*e.g.* average values) and not individual-centered. Therefore, our model allowed to quantify and characterise the potential interactions, providing that relevant confounding factors are taken into account. It provides avenues and opportunities to deepen thinking on the interaction modelling. This method responds to current needs by taking into account multi-pathogen relationships, which will allow the development of better controls and preventive health care. By identifying pairs of potentially interacting species [57]–[60], it furthermore will clarify fundamental processes of organism assemblages.

## Supporting Information

Table S1 Variation of the α risk under H0 of test based on our probabilistic model, according to different number of individuals and different pathogen prevalences. 1000 simulations were performed to create a simulated data set. The numbers of individuals of different states of infection were drawn at random from a multinomial distribution. The model parameters were fixed *a priori*: (p_i_,q_i_) ε {0.10,0.20,0.30,0.40,0.50,0.60,0.70,0.80,0.90} under the null hypothesis (W_a_ = W_A_ = 1), for a range from 200 to 1000 individuals. The estimated α risk can thus be compared to the theoretical value defined *a priori* (0.05).
(XLS)

Table S2 Variation of the α risk under H0 of Chi-square test of independence for juveniles and for adults, according to different number of individuals and different pathogen prevalences. 1000 simulations were performed to create a simulated data set. The numbers of individuals of different states of infection were drawn at random from a multinomial distribution. The model parameters were fixed *a priori*: (p_i_,q_i_) ε {0.10,0.20,0.30,0.40,0.50,0.60,0.70,0.80,0.90} under the null hypothesis (W_a_ = W_A_ = 1), for a range from 200 to 1000 individuals. The estimated α risk can thus be compared to the theoretical value defined *a priori* (0.05).
(XLS)

Table S3 Evaluation of the power of the test (1 - β) under H1 for different weights of infection for test based on our probabilistic model. 1000 simulations were performed to create a simulated data set. The numbers of individuals of different states of infection were drawn at random from a multinomial distribution. The model parameters were fixed *a priori*: (p_i_,q_i_) ε {0.10,0.20,0.30,0.40,0.50,0.60,0.70,0.80,0.90} under different alternative hypotheses with W_a_ ε [0.5, 1.5] and W_A_ ε [0.5, 1.5], for a range from 200 to 1000 individuals.
(XLS)

Table S4 Evaluation of the power of the test (1 - β) under H1 for different weights of infection for Chi-square test of independence for juveniles and for adults. 1000 simulations were performed to create a simulated data set. The numbers of individuals of different states of infection were drawn at random from a multinomial distribution. The model parameters were fixed *a priori*: (p_i_,q_i_) ε {0.10,0.20,0.30,0.40,0.50,0.60,0.70,0.80,0.90} under different alternative hypotheses with W_a_ ε [0.5, 1.5] and W_A_ ε [0.5, 1.5], for a range from 200 to 1000 individuals.
(XLS)

Table S5 Comparison of observed and estimated of different states of infection for the final model. The total sample size was 119 for juveniles (A) and 324 for adults (B).
(XLS)

Functions S1 **Functions to estimate the model probabilities.**
(TXT)
